# Adapted Judo as a Multidimensional Intervention: Effects on Physical Fitness and Psychosocial Well-Being in Adolescents with Down Syndrome

**DOI:** 10.3390/healthcare14010081

**Published:** 2025-12-30

**Authors:** Borja Suarez-Villadat, Mario Montero, Sonia Montero, Adrián López-García, Ariel Villagra

**Affiliations:** 1 Department of Physical Therapy, Occupational Therapy, Rehabilitation and Physical Medicine, Health Sciences Faculty, Universidad Rey Juan Carlos, 28922 Madrid, Spain; 2 Faculty of Sport Science, Pablo Olavide University, 41013 Sevilla, Spain; mrmntr@uax.es (M.M.); smontero@uax.es (S.M.); 3 Faculty of Biomedical and Health Sciences, Alfonso X el Sabio University, 28691 Madrid, Spain; 4 Sport Sciences Research Centre, Universidad Rey Juan Carlos, 28942 Madrid, Spain; adrnlpz@uax.es; 5 Department of Physical Education, Sport and Human Movement, Autonomous University of Madrid, 28049 Madrid, Spain; ariel.villagra@uam.es

**Keywords:** adapted judo, Down syndrome, physical fitness, psychosocial well-being, inclusive physical activity

## Abstract

**Highlights:**

**What are the main findings?**

- Adapted judo significantly improved physical fitness components (strength, balance, and flexibility) in adolescents with Down syndrome.
- The intervention also enhanced psychosocial well-being, including self-esteem and social interaction.

**What are the implications of the main findings?**

- Adapted judo can be considered a multidimensional strategy for promoting health and inclusion in adolescents with Down syndrome.
- These findings support the integration of adapted martial arts into physical education and therapeutic programs.

**Abstract:**

**Background/Objectives**: Adolescents with Down syndrome often present limitations in physical fitness and psychosocial well-being, which can affect their health and social inclusion. Adapted physical activity programs, such as martial arts, may offer multidimensional benefits. This study aimed to analyze the effects of an adapted judo intervention on physical fitness and psychosocial outcomes in adolescents with Down syndrome. **Methods**: A quasi-experimental design was applied with 43 adolescents diagnosed with Down syndrome, allocated to a control group (n = 19) and an intervention group (n = 24). Participants in the intervention group completed a 24-week adapted judo program. Physical fitness was assessed through standardized tests for strength, balance, and flexibility, while psychosocial well-being was evaluated using validated questionnaires on self-esteem and social interaction. Pre- and post-intervention comparisons were conducted using appropriate statistical analyses. **Results**: Participants showed significant improvements in physical fitness components, particularly in muscular strength and balance (*p* < 0.05). Flexibility also increased, although to a lesser extent. Psychosocial measures revealed enhanced self-esteem and greater perceived social interaction, indicating positive effects beyond physical health. **Conclusions**: Adapted judo appears to be an effective multidimensional intervention for adolescents with Down syndrome, promoting both physical and psychosocial benefits. These findings support the inclusion of adapted martial arts in physical education and therapeutic programs aimed at fostering health and social participation in this population.

## 1. Introduction

Down syndrome (DS) is the most common genetic cause of intellectual disability worldwide, resulting from the presence of an extra copy of chromosome 21 [1,2]. Its prevalence is approximately 1 in 1000 live birth [1]. Individuals with DS exhibit distinctive phenotypic and functional characteristics, including muscle hypotonia, ligamentous laxity, short stature, abdominal obesity, and reduced muscle mass, which negatively affect motor performance and autonomy [3,4]. These limitations are associated with lower scores in strength, endurance, agility, and balance compared to peers without disabilities or with other intellectual disabilities [5,6]. Additionally, prolonged sedentary behavior increases the risk of metabolic and cardiovascular disorders, reinforcing the need for preventive strategies [7,8].

Structured physical activity has been widely recognized as an effective approach to mitigate these limitations, as evidence indicates that exercise programs improve body composition, reduce body fat percentage, increase muscular strength, and enhance cardiorespiratory fitness in adolescents with DS [9,10]. However, higher activity levels do not always translate into proportional improvements in physical fitness, highlighting the importance of intervention quality and specificity [11]. Within this framework, modalities such as swimming, resistance training, and aerobic exercise have demonstrated positive effects on health-related fitness and body composition, including reductions in adiposity and gains in muscular strength [9,10,12,13]. Despite these benefits, adherence to conventional exercise programs can be challenging for adolescents with DS due to motivational barriers, limited accessibility, and lack of inclusive pedagogical strategies [14].

Among structured interventions, adapted martial arts particularly judo stand out for their dual contribution to physical and psychosocial development. Judo combines motor skill acquisition with educational values such as respect, discipline, and cooperation [5]. Research suggests that adapted judo programs can improve muscular strength, dynamic balance, and body composition while fostering psychosocial outcomes such as self-esteem, emotional regulation, and social interaction [4,15,16,17]. Beyond physical benefits, these programs promote inclusion and emotional well-being, reducing social isolation and enhancing self-confidence [18,19,20]. Furthermore, martial arts-based interventions have been associated with improvements in executive functioning and behavioral regulation in populations with developmental disorders, suggesting a broader impact on cognitive and socioemotional domains. Judo offers unique benefits for proprioception and intermuscular coordination compared to swimming or resistance training, due to its emphasis on dynamic balance, multi-planar movements, and controlled falls that stimulate neuromuscular responses [21].

Despite these promising findings, evidence on the long-term effects of adapted judo on psychosocial variables and motor learning in adolescents with DS remains limited, particularly in controlled interventions. Therefore, the present study aims to evaluate the impact of a 24-week adapted judo program on physical fitness, agility, and psychosocial outcomes in adolescents with DS.

## 2. Materials and Methods

### 2.1. Study Design and Setting

This study employed a quasi-experimental design with random allocation to intervention (IG) and control (CG) groups. The intervention consisted of a 24-week adapted judo program implemented during the 2023/2024 academic year in a Special Education School in Madrid, Spain. Sessions were conducted in the school gymnasium equipped with tatami mats and safety materials, under the supervision of two certified judo instructors who were blinded to the study objectives. Random allocation was performed using a concealed assignment and block randomization method to ensure balanced distribution between groups [22]. Six subgroups were created, each containing two participants from the CG and two from the IG. The sequence of subgroup assignments was determined using random numbers and sealed envelopes, and the allocation was carried out by a physical therapist not involved in the study [23]. These procedures represent methodological strengths that reduce bias and potential confounding factors.

### 2.2. Recruitment

Participants were recruited from a Special Education School in Madrid during the 2023/2024 academic year. Information sessions were organized with school staff, students, and parents to explain the study objectives, procedures, and safety measures. Written informed consent was obtained from parents or legal guardians prior to enrollment.

Initially, 59 students were screened for eligibility. Seven adolescents with intellectual disabilities but without DS were excluded to maintain sample homogeneity, although they were initially considered to avoid discrimination within the school setting. Three participants were removed due to acute illness, three did not complete the baseline physical fitness assessments, and two declined participation after recruitment.

### 2.3. Participants

The final sample consisted of 43 adolescents with DS aged 15 to 17 years (SD ± 0.87), including 16 females and 27 males. The control group comprised 19 participants (16.1 ± 1.30 years; 9 females, 10 males), while the intervention group included 24 participants (16.6 ± 0.97 years; 7 females, 17 males).

### 2.4. Inclusion Criteria

Participants were required to meet the following conditions: (i) A confirmed diagnosis of DS provided by a certified physician; (ii) Enrollment in a Special Education School to ensure a homogeneous educational context; (iii) No participation in any structured exercise training program during the previous three months; (iv) An intelligence quotient greater than 35, assessed using the Full Scale Intelligence Quotient (FSIQ) [24]; (v) Ability to follow basic instructions and participate safely in physical activities, as determined by the supervising educator. To minimize performance bias, the two judo instructors involved in the intervention were not informed of the study objectives; (vi) Completion of the Physical Activity Readiness Questionnaire (PAR-Q) and provision of written informed consent from parents or legal guardians, together with medical clearance (provided by the school’s healthcare professionals) confirming absence of cardiovascular conditions (e.g., congenital heart disease) and joint disorders such as atlantoaxial instability, patellar instability or recurrent patellar dislocation due to the increased risk of cervical spine injury during physical activity [25,26].

### 2.5. Exclusion Criteria

Participants were excluded if they presented any of the following: (i) Severe sensory impairments (visual or auditory) or physical injuries that could compromise safe participation; (ii) Neurological conditions such as encephalopathy or epilepsy, or congenital cardiopulmonary disease; (iii) Severe functional limitations preventing execution of basic motor tasks; (iv) Absence of written informed consent from parents or legal guardians, or failure to complete the Physical Activity Readiness Questionnaire; (v) Intellectual disability classified as severe or profound, based on parental report and clinical guidelines [27,28].

### 2.6. Intervention. Adapted Judo Program

The intervention program lasted 24 weeks and followed a structured pedagogical sequence integrating adapted judo techniques with targeted objectives across three core dimensions: physical fitness enhancement, body composition optimization, and socioemotional development. The program was designed by three black belt judokas, based on evidence-based frameworks from recent research on adapted martial arts and inclusive physical education [4,5,20]. The intervention program lasted 24 weeks and consisted of three weekly sessions, each lasting 90 min. Sessions were conducted in the school gymnasium under the supervision of two black belt judokas (distinct from the program designers). These studies informed the progression, safety protocols, and methodological principles applied throughout the intervention. Each phase was structured to simultaneously address emotional competencies, motor skills, and functional parameters, in line with adapted physical activity principles and inclusive education guidelines.

Weeks 1–2: The initial phase focused on creating a safe and predictable environment through formal dojo (training hall) introduction, ritualized greetings, and basic locomotor drills. These activities promoted group cohesion, activated general mobility, and allowed baseline observations of postural alignment.

Weeks 3–4: Basic falling techniques (ukemi) and paired exercises were introduced to improve intermuscular coordination, body schema activation, and emotional recognition, while enhancing joint mobility and functional stability.

Weeks 5–7: The program emphasized impulse control and emotional regulation through grip techniques (kumi-kata) and foundational stances requiring postural control and functional strength, contributing to muscular endurance and improved body tone.

Weeks 8–9: Emotional and motor responsibility were reinforced through opposition-based movements and cooperative tasks, stimulating shared decision-making and improving aerobic capacity via dynamic activities.

Weeks 10–11: Self-confidence and self-esteem were strengthened through adapted projection techniques with assisted execution, enabling assessment of lower-limb power and technical performance under controlled conditions.

Weeks 12–14: Initiative and autonomy were fostered through symbolic attack and defense games with flexible rules, involving rapid directional changes, agility, and real-time decision-making, thereby improving reaction speed and global coordination.

Weeks 15–17: Motor control and emotional self-regulation were consolidated through predictable technical sequences with role alternation, enhancing anticipatory skills, motor memory, and dynamic stability.

Weeks 18–21: Adaptability and cognitive flexibility were addressed through controlled sparring (randori) under modified rules, requiring aerobic endurance, frustration tolerance, and cardiovascular efficiency.

Weeks 22–23: Empathy and cooperative engagement were promoted through paired tasks involving weight transfer and bilateral coordination, assessing functional interaction and collaborative capacity.

Week 24: The final phase integrated all learning outcomes through a structured demonstration, closing ritual, and symbolic greeting, complemented by guided self-assessment and verbal reflection, enabling evaluation of socioemotional progress, physical fitness improvements, and observable changes in body composition.

Participants in the control group continued with their usual school schedule, which included standard physical education activities typically provided in the special education curriculum. These activities were non-structured and focused on basic mobility and recreational tasks, without any adapted judo or targeted exercise program.

### 2.7. Adherence Monitoring

Adherence was defined as attendance at ≥80% of the scheduled sessions (a minimum of 58 out of 72 sessions during the 24-week program), consistent with previous recommendations for exercise interventions in individuals with intellectual disabilities [29,30]. Attendance was recorded at each session by school healthcare professionals and judo instructors using standardized checklists, following protocols reported in adapted physical activity programs [31]. Participants missing more than 20% of sessions or failing to complete baseline or post-intervention assessments were excluded from the final analysis. Reasons for exclusion included acute illness or withdrawal of consent, as described in previous studies on neuromuscular interventions in adolescents with DS [14,32].

### 2.8. Data Measurements

#### 2.8.1. Assessment of Health-Related Physical Fitness

Body composition assessments were conducted by health professionals, while physical fitness evaluations were carried out by sports specialists. Health-related physical fitness indicators including BMI, waist and hip circumferences, percentage of body fat, handgrip strength, standing long jump, Timed Up and Go test, deep trunk flexibility, 10 Timed-Stand Test, 30 s sit-up, and the 6 min walk test were assessed following the standardized procedures described in previous research [23,33]. These protocols have been validated for use in adolescents with DS in multiple studies [33,34]. Additionally, maximal strength was evaluated through one-repetition maximum (1RM) tests for chest press and leg press exercises, applying a recognized 1RM assessment protocol. The reliability of these tests has been documented in prior investigations [35], showing high reproducibility (r = 0.89) and no systematic variation after three weeks of application in adults with neurological conditions [28].

All measurements were performed at baseline and post-intervention, with participants barefoot and wearing light clothing. Height and weight were recorded using an electronic scale with 0.1 kg precision and a telescopic stadiometer accurate to 1 mm (SECA 701-202, Hamburg, Germany). BMI was calculated as weight divided by height squared (kg/m^2^).

Anthropometric variables included waist and hip circumferences. Waist circumference was measured at the midpoint between the iliac crest and the lowest rib using a non-elastic tape [36]. Hip circumference was taken at the most prominent point of the buttocks, ensuring the tape lightly touched the skin without compression [37]. Body fat percentage was estimated through skinfold thickness using the Slaughter et al. equation, validated for adolescents with DS [38,39].

Muscle strength was assessed using a one-repetition maximum (1RM) protocol for seated chest press and leg press. These tests have demonstrated high reliability (r = 0.89) and stability over time in populations with neurological impairments [28,35]. Handgrip strength was measured with a handheld dynamometer (TKK 5101 Grip D; Takey, Tokyo, Japan) in a seated position, a method shown to yield consistent results compared to standing [33,34]. Lower-body power was evaluated using the standing long jump test, measuring the distance from the take-off line to the rearmost landing point [34]. Agility and dynamic balance were assessed with the Timed Up and Go test, where participants walked 3 m and returned as quickly as possible; the best of two trials was recorded [40,41].

Flexibility was measured using the Deep Trunk Flexibility test, requiring participants to reach behind their legs and slide a millimeter bar cursor on a standardized bench [42]. Muscular endurance was assessed through the Timed-Stands Test (10 squats as fast as possible) and the 30 s Sit-Up Test, following protocols adapted for individuals with DS [43,44].

Finally, cardiorespiratory endurance was evaluated using the 6-Minute Walk Test, where participants walked as far as possible on level ground within six minutes; the total distance was recorded [33,45].

#### 2.8.2. Agility Tasks

The Bruininks-Oseretsky Test of Motor Proficiency, 2° Edition (BOT-2) is a standardized instrument designed to assess fine and gross motor skills in individuals aged 4 to 21 years, primarily within the school-age population. It is suitable for participants with a wide range of motor abilities, from typical development to mild or moderate impairments. The assessment comprises four subtests organized into eight composite scores, targeting domains such as stability, mobility, strength, coordination, and object manipulation. BOT-2 is widely employed by professionals to evaluate psychomotor development and identify motor difficulties [46].

Evidence supports the reliability and construct validity of BOT-2 in children with intellectual disabilities, demonstrating strong internal consistency, test–retest reliability, and responsiveness [47]. In the present study, the strength and agility composite were utilized, which includes four specific subtests: push-ups, sit-ups, wall sit, and V-up.

#### 2.8.3. Psychosocial Variables Assessment

In practical settings, the evaluation of psychosocial abilities in individuals with intellectual disabilities often relies on subjective judgments of social behavior [48]. To overcome this limitation, the present study employed a standardized instrument specifically developed for assessing psychosocial characteristics [49]. Peric’s questionnaire comprises 51 items distributed across four domains: aggression, attention disorders, anxiety-depression, and social problems.

The scoring process required the participation of a certified special educator independent from the research team along with input from the adolescent’s parent or legal guardian. Following these steps, a structured conversation with the participant was conducted in the presence of the legal guardian to guarantee ethical compliance and accurate interpretation of responses [48,50].

##### Familiarization

Prior to the 24-week intervention, a one-week familiarization phase was conducted to ensure participants understood the procedures and requirements of the physical fitness and agility assessments. During this period, students were introduced to each test included in the evaluation protocol, received detailed explanations, and practiced the movements under supervision. This preparatory stage aimed to minimize anxiety, improve compliance, and ensure that all participants could perform the physical fitness and agility tests correctly and safely during the official assessment sessions. Similar procedures have been recommended for individuals with DS to ensure valid and reliable test results [32,51]

#### 2.8.4. Ethical Approval

The study protocol complied with the principles of the Declaration of Helsinki [52] and received approval from the Bioethics Committee of the Autonomous University of Madrid. Participants and their families were provided with comprehensive information about the study, and written informed consent was obtained prior to participation.

#### 2.8.5. Statistical Analysis

A mixed-design analysis of variance (ANOVA) with repeated measures was applied to examine the effects of the adapted judo program on health-related physical fitness, agility, and psychosocial variables. The model included two factors: time (pretest vs. posttest) as the within-subjects factor and group (control vs. intervention) as the between-subjects factor. This approach allowed testing for main effects and interaction effects between time and group [53]. Normality assumptions were verified through skewness and kurtosis values, which indicated an approximately normal distribution for all variables. When significant interaction effects were detected, Bonferroni-adjusted pairwise comparisons were conducted to identify differences between groups at post-intervention. Descriptive statistics (mean and standard deviation) were computed for all variables. Effect sizes were calculated using partial eta squared (η^2^) to determine the magnitude of observed differences, interpreted according to conventional thresholds (small = 0.01, medium = 0.06, large = 0.14) [54]. The level of significance was set at *p* < 0.05. Although a formal sample size calculation was not performed prior to data collection due to setting constraints, a post hoc power analysis was conducted using G*Power 3.1 based on observed large effect sizes (η^2^ = 0.24–0.29) and *p* = 0.05, indicating sufficient power (>0.94) for the main outcomes [55].

## 3. Results

**Figure 1:**
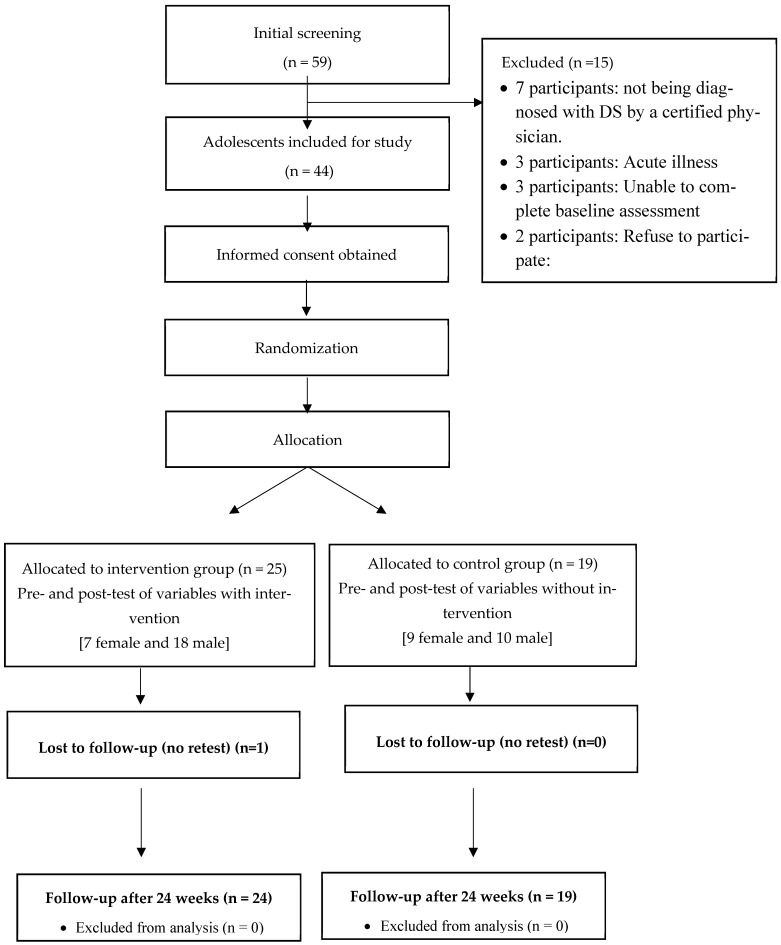
Participants’ flow diagram.

Figure 1 illustrates the flow diagram showing the random allocation of adolescents to either the CG or IG. Table 1 presents baseline participant characteristics. Descriptive statistics (mean and standard deviation) for all variables were obtained through pretest and posttest assessments. Health-related physical fitness outcomes for both groups are summarized in Table 2, while agility and psychosocial parameters are detailed in Table 3. Between-group comparisons were conducted at the post-intervention evaluation point after 24 weeks of the adapted judo program. Significant differences were observed in several body composition, physical fitness, agility, and psychosocial variables. Body composition: Waist circumference showed significant reductions in the IG compared to the CG (*p* = 0.043; η^2^ = 0.18). Hip circumference also differed significantly between groups (*p* < 0.001; η^2^ = 0.23), as did body fat percentage (*p* = 0.009; η^2^ = 0.20). BMI did not show significant differences between groups (*p* > 0.05). Physical fitness: Handgrip strength improved significantly more in the IG (*p* < 0.001; η^2^ = 0.25), as did chest press 1RM (*p* = 0.007; η^2^ = 0.21) and leg press 1RM (*p* < 0.001; η^2^ = 0.28). Muscular endurance measured by the 30 s sit-up test also showed a significant improvement (*p* < 0.001; η^2^ = 0.27) and 10 Timed-Stand Test (*p* = 0.025; η^2^ = 0.27). No significant differences were found in the standing long jump (*p* > 0.05), while the Timed Up and Go test showed a moderate improvement (*p* = 0.046; η^2^ = 0.15). Agility: The IG achieved higher scores in the shuttle run (*p* = 0.001; η^2^ = 0.24), one-legged side hop (*p* = 0.008; η^2^ = 0.23) and two-legged side hop (*p* = 0.029; η^2^ = 0.27), while the one-legged stationary jump showed marginal differences (*p* = 0.010). The total agility score reflected a significant overall improvement (*p* = 0.028). Psychosocial variables: Significant reductions were observed in aggression (*p* = 0.041; η^2^ = 0.29), attention disorders (*p* = 0.034; η^2^ = 0.26), anxiety-depression (*p* = 0.031; η^2^ = 0.27), and social problems (*p* = 0.007; η^2^ = 0.30) in the IG, indicating a positive impact of the adapted judo program on emotional and social well-being. Mixed-design ANOVA with repeated measures; Bonferroni-adjusted pairwise comparisons. Values are expressed as mean ± standard deviation. *p*-values indicate significance within groups and interaction effects (*p* = 0.05).

## 4. Discussion

The primary aim of this study was to evaluate the effects of a 24-week adapted judo program on physical fitness and psychosocial well-being in adolescents with DS. In line with this objective, the findings revealed significant improvements in muscular strength, endurance, and agility, as well as reductions in waist and hip circumference and body fat percentage. Additionally, psychosocial outcomes such as aggression, anxiety, and attention problems showed marked decreases. These results indicate that adapted judo can serve as an effective multidimensional intervention to promote health and social inclusion in this population.

The adapted judo program produced significant reductions in waist and hip circumference and body fat percentage among adolescents with DS, while BMI did not exhibit statistically significant changes. This outcome is consistent with previous research emphasizing the limited sensitivity of BMI in detecting body composition changes in individuals with DS due to their unique anthropometric characteristics, such as shorter stature and higher fat proportion [56]. Structured physical activity interventions can improve body composition without altering BMI, reinforcing the need for more precise indicators like waist circumference and body fat percentage when evaluating health outcomes [43].

The reduction in central adiposity observed in our study is clinically relevant because abdominal obesity is strongly associated with metabolic and cardiovascular risk factors in adolescents with DS [7,8]. These findings align with Aksovic et al. [57], who demonstrated improvements in body composition following adapted exercise programs, highlighting the importance of individualized and supervised interventions. The observed decrease in waist and hip circumference suggests a redistribution of body fat, which may contribute to reducing obesity-related complications and improving overall metabolic health.

It is noteworthy that the intervention was implemented in a school setting, underscoring the feasibility of integrating adapted physical activity programs into educational environments. This approach is particularly important given that adolescents with DS typically exhibit lower levels of physical activity compared to peers without disabilities, increasing vulnerability to obesity and related comorbidities [58]. Incorporating adapted judo into school curricula can provide structured opportunities for movement that not only improve physical health but also foster inclusion and social engagement. Despite these positive outcomes, the absence of BMI changes warrants discussion. BMI is a crude measure that does not differentiate between fat mass and lean mass, making it less suitable for populations with atypical growth patterns. Hilgenkamp et al. [59] previously noted that BMI fails to capture subtle improvements in body composition, particularly when interventions increase muscle mass while reducing fat mass. Therefore, future studies should prioritize more sensitive measures, such as skinfold thickness or bioelectrical impedance analysis, to accurately assess changes in body composition. Another relevant consideration is the potential influence of uncontrolled dietary intake. Although the intervention focused exclusively on physical activity, caloric intake was not monitored due to logistical constraints within the school setting. This limitation may have introduced variability in body composition outcomes, as energy balance plays a critical role in fat reduction. Future research should incorporate nutritional assessments or standardized dietary guidelines to minimize this confounding factor.

The adapted judo program produced significant improvements in multiple components of physical fitness, including handgrip strength, maximal strength in upper and lower limbs (assessed through 1RM tests), muscular endurance, and agility. These results corroborate findings from Shields et al. [60] who documented the positive effects of progressive resistance and adapted exercise programs on strength development in adolescents with DS. Enhanced muscular strength and endurance are particularly relevant for this population, as they contribute to functional independence and improved quality of life [61].

Compared to other physical activity modalities, martial arts-based interventions appear to offer superior benefits in terms of strength, coordination, and motor control. Descamps et al. [62] suggested that the multifaceted nature of judo combining dynamic movements, cognitive engagement, and social interaction creates an enriched environment for motor learning. Our findings on agility and coordination are consistent with those reported by Federici et al. [21], who emphasized that martial arts programs enhance intermuscular coordination and proprioceptive awareness, both of which are critical for autonomy and participation in daily activities.

The improvement in agility observed in this study is particularly relevant due to its association with functional mobility and fall prevention. Individuals with DS often experience hypotonia and ligamentous laxity, which compromise balance and increase fall risk [5,6]. Incorporating exercises that challenge dynamic stability and reaction time, such as those in adapted judo, may mitigate these risks and promote safer movement patterns. Gains in maximal strength and muscular endurance further enhance tolerance to physical effort, enabling adolescents to perform daily tasks with greater ease and confidence. These improvements in strength and agility have broader implications for health and functionality. Increased muscular strength supports better postural control, gait stability, and reduces musculoskeletal injury risk. Similarly, improved agility facilitates participation in recreational activities and sports, promoting social inclusion and psychological well-being [63]. Overall, these findings underscore the potential of adapted judo as a comprehensive intervention addressing both physical and psychosocial dimensions of health [63,64,65].

The pedagogical structure of the program likely contributed to these outcomes by using a progressive approach that combined technical drills with cooperative and competitive activities, sustaining engagement and motivation. Previous studies emphasize the role of enjoyment and social interaction in adherence to physical activity programs among individuals with intellectual disabilities [66,67]. In this context, adapted judo not only improved physical fitness but also fostered a sense of belonging and accomplishment, supporting long-term participation.

Beyond physical benefits, the program produced significant psychosocial improvements, including reductions in aggression, attention problems, anxiety-depression, and social difficulties. These findings align with evidence on the positive impact of physical activity on emotional well-being and social functioning in individuals with intellectual disabilities [49]. Similar effects have been reported in adapted exercise interventions, improving self-regulation and reducing disruptive behaviors [68]

Our results are consistent with studies showing martial arts programs enhance social skills and reduce maladaptive behaviors in populations with developmental disorders [69,70]. Differences from Harwood-Gross et al. [71], who found no changes in aggression among neurotypical adolescents, may reflect the unique behavioral profiles of individuals with DS and the structured, supportive nature of adapted programs.

The psychosocial benefits observed also agree with reviews highlighting the effectiveness of interventions combining physical and cognitive challenges in promoting self-esteem and social competence [31]. By fostering cooperation, respect, and mutual support, adapted judo creates a social context that reduces isolation and promotes holistic development. Furthermore, reductions in anxiety and depressive symptoms have implications for mental health promotion, as adolescents with DS are at increased risk for emotional disorders. Structured physical activity programs incorporating emotional regulation strategies may serve as preventive interventions, reinforcing resilience and coping skills. These findings support multidisciplinary approaches integrating physical education, psychology, and special education to optimize outcomes [72,73,74].

Within the limitations of this study, adapted judo programs appear to offer promising benefits for physical fitness and psychosocial well-being in adolescents with DS. Integrating these programs into school-based physical education could promote health, social inclusion, and emotional regulation. Structured, progressive methodologies combining technical instruction with cooperative and playful activities may enhance adherence and motivation. Community-based implementation can provide accessible opportunities for physical activity, reduce sedentary behavior, and foster life skills such as self-control, empathy, and resilience.

Further research is needed to confirm these findings and strengthen generalizability. Future studies should include larger, gender-balanced samples and adopt longitudinal designs to assess sustainability. Comparative analyses of different adapted physical activity modalities (e.g., judo, swimming, soccer) could identify the most effective approaches. Incorporating objective measures of adherence, motivation, and cognitive functioning would clarify underlying mechanisms. Exploring digital tools and remote monitoring could improve accessibility, especially in underserved areas.

### Limitations

This study has limitations, small sample size, unequal gender distribution, and implementation in a single educational center, which may restrict external validity. The absence of long-term follow-up limits conclusions on sustainability. Adherence and motivation were not objectively monitored, and dietary intake was uncontrolled, potentially influencing body composition outcomes. Future research should include nutritional assessments or standardized dietary guidelines to minimize this confounding factor.

A key limitation of this study is that adherence was assessed exclusively through attendance records, without incorporating indicators of engagement, motivation, or enjoyment. Recent research in populations with DS and intellectual disabilities has identified this gap as a methodological weakness. For example, D’Amours et al. [75] reported insufficient fidelity and sustainability measures in school-based interventions for individuals with disabilities, emphasizing the lack of engagement metrics. Similarly, Terra et al. [76] highlighted motivation and encouragement as common barriers to participation in adolescents with intellectual disabilities, while Kim et al. [77] noted the absence of validated tools to capture motivational aspects in inclusive programs. Qualitative studies also confirm that teachers often lack strategies to foster motivation among students with DS [78]. To address this limitation, future research should integrate validated instruments such as the Intrinsic Motivation Inventory and its adaptations for physical education, as well as enjoyment scales like ENJOY [79], to provide a more comprehensive understanding of participant engagement.

A major limitation of this study is the absence of an a priori sample size calculation, which restricts the ability to ensure optimal statistical power before data collection. This issue is particularly critical in research involving individuals with DS because the population is small and heterogeneous, making recruitment challenging and increasing the risk of underpowered analyses. The lack of prospective calculation may increase the risk of Type II error and limit the generalizability of findings, as highlighted in previous research on exercise interventions for individuals with DS [61]. Future studies should incorporate formal sample size estimation to strengthen methodological rigor and ensure adequate power for detecting clinically meaningful differences.

Finally, psychosocial outcomes were assessed using standardized questionnaires completed by educators and parents, which, although validated, may be subject to reporting bias. Including objective behavioral measures or multi-informant approaches could strengthen the reliability of future research.

In summary, the findings of this study underscore the multifaceted benefits of adapted judo for adolescents with DS, encompassing improvements in body composition, physical fitness, and psychosocial well-being. These outcomes highlight the value of structured, inclusive programs that integrate motor, cognitive, and emotional components within educational and community settings. By promoting functional independence, emotional regulation, and social participation, adapted judo emerges as a comprehensive intervention aligned with current recommendations for holistic health promotion in populations with intellectual disabilities. Future efforts should focus on scaling these programs, ensuring accessibility, and exploring long-term impacts to consolidate their role as a cornerstone of inclusive physical education and preventive mental health strategies.

## 5. Conclusions

The present study demonstrates that a 24-week adapted judo program produces significant improvements in physical and psychosocial outcomes among adolescents with intellectual disabilities, including those with DS. Notable gains were observed in muscular strength, endurance, agility and functional performance, alongside reductions in waist and hip circumference, body fat percentage, and psychosocial difficulties such as aggression, anxiety, and attention problems. These findings provide preliminary evidence that adapted judo may be a feasible and motivating intervention to enhance health and social participation, warranting further research with larger and more diverse samples. Despite limitations related to sample size and generalizability, the findings highlight the potential of integrating adapted martial arts into educational and community settings to promote comprehensive physical and emotional development in youth with intellectual disabilities.

## Figures and Tables

**Table 1:** Baseline participants characteristics.

Psychophysical Characteristics	Groups Mean (SD)	
	Control Group	Intervention Group
N	19	24
Age (years)	16.1 (1.30)	16.6 (0.97)
Weight (kg)	77.49 (6.64)	73.17 (9.08)
Height (cm)	161.4 (7.20)	159.4 (4.30)
FSIQ (score)	55.94 (9.09)	58.42 (8.17)
Level of perceived ID, *n* (%) ^a^		
Mild	14	21
Moderate	5	3

Abbreviations; SD: standard deviation; ID: intellectual disability. ^a^ Or *n* and percentage when indicated. FSIQ, Wechsler Intelligence Scale for Children-V Full Scale Intelligence Quotient.

**Table 2:** Health-related physical fitness. Pretest and posttest after 24 weeks.

Outcome	Control Pre (Mean ± SD)	Control Post (Mean ± SD)	*P* (Within CG)	Intervention Pre (Mean ± SD)	Intervention Post (Mean ± SD)	*P* (Within IG)	*P* (Interaction)
**Body composition**							
BMI (kg/m^2^)	29.66 (4.85)	30.85 (5.42)	0.214	28.89 (3.92)	27.92 (3.32)	0.083	>0.05
Waist circumference (cm)	80.35 (6.36)	81.76 (7.23)	0.312	81.67 (10.50)	74.36 (9.71)	0.043	0.043
Hip circumference (cm)	92.51 (5.24)	91.73 (5.68)	0.401	90.15 (9.65)	86.94 (8.42)	<0.001	<0.001
Body fat (%)	31.46 (5.02)	32.98 (4.08)	0.278	31.89 (11.97)	29.90 (13.16)	0.009	0.009
**Physical fitness**							
Handgrip strength (kg)	19.16 (3.36)	20.23 (2.40)	0.412	19.67 (2.40)	25.42 (2.09)	<0.001	<0.001
Chest press 1rm (kg)	30.86 (2.89)	32.58 (2.67)	0.368	31.10 (3.36)	35.62 (3.34)	0.007	0.007
Standing long jump (cm)	75.93 (6.79)	69.71 (6.23)	0.032	71.94 (3.76)	70.87 (5.85)	0.521	>0.05
Leg press 1rm (kg)	75.47 (6.73)	73.91 (7.71)	0.521	77.80 (8.05)	83.50 (10.74)	<0.001	<0.001
**Motor fitness**							
Timed up and go (s)	4.87 (1.90)	5.02 (2.05)	0.401	4.93 (0.90)	4.21 (1.61)	0.046	0.046
**Musculoskeletal fitness**							
Deep trunk flexibility (cm)	29.54 (3.61)	31.89 (4.46)	0.083	30.63 (2.79)	37.10 (2.29)	0.083	>0.05
10 timed-stand test (s)	17.11 (1.53)	17.25 (1.88)	0.278	21.20 (3.84)	18.01 (4.05)	0.025	0.025
30 s sit-up (repetitions)	12.78 (2.35)	11.31 (2.24)	0.312	13.85 (4.23)	17.32 (5.61)	<0.001	<0.001
**Cardiorespiratory fitness**							
6 min walk test (m)	426.20 (17.61)	439.26 (21.16)	0.214	413.63 (15.28)	421.95 (20.34)	0.083	>0.05

Mixed-design ANOVA with repeated measures; Bonferroni-adjusted pairwise comparisons. Values are expressed as mean ± standard deviation. *p*-values indicate significance within groups and interaction effects (*p* = 0.05). CG (control group); IG (intervention group).

**Table 3:** Descriptive statistics of pretest and posttest in each group for agility, and psychosocial variables.

Outcome	Control Pre (Mean ± SD)	Control Post (Mean ± SD)	*P* (Within CG)	Intervention Pre (Mean ± SD)	Intervention Post (Mean ± SD)	*P* (Within IG)	*P* (Interaction)
**Agility**							
Shuttle run	5(1.76)	5(1.26)	0.754	5(0.99)	6(1.74)	0.042	0.001
Stepping sideways	3(0.94)	3(0.88)	0.408	3(0.94)	3(1.37)	0.781	0.354
One-legged stationary jump	3(2.09)	3(1.99)	0.869	4(1.43)	5(1.26)	0.044	0.010
One-legged side hop	4(1.98)	3(1.41)	0.748	5(1.57)	7(1.66)	0.048	0.008
Two-legged side hop	4(1.73)	4(1.65)	0.170	4(0.89)	6(1.02)	0.027	0.029
Total agility score	12(9.09)	11(10.67)	0.695	10(7.13)	12(1.24)	0.003	0.028
**Psychosocial variables**							
Aggression	55.78(12.02)	56.02(11.47)	0.678	53.33(10.47)	44.21(11.72)	0.042	0.041
Attention disorders	52.62(9.31)	53.88(10.22)	0.196	55.45(8.43)	47.64(10.92)	0.005	0.034
Anxiety and depression	50.91(12.61)	52.19(13.38)	0.105	49.18(10.70)	41.10(11.23)	0.015	0.031
Social problems	52.38(13.47)	54.85(11.82)	0.163	54.45(9.98)	49.22(10.69)	0.020	0.007

Mixed-design ANOVA with repeated measures; Bonferroni-adjusted pairwise comparisons. Values are expressed as mean ± standard deviation. *p*-values indicate significance within groups and interaction effects (*p* = 0.05). CG (control group); IG (intervention group).

## Data Availability

The data presented in this study are available on request from the corresponding author. Due to ethical considerations and privacy concerns related to participants with disabilities, the data cannot be shared publicly.

## Abbreviations

The following abbreviations are used in this manuscript:

DS	Down syndrome
BMI	Body mass index
SD	Standard Deviation
*p*	*p*-value
